# Understanding barriers to the provision of hand hygiene products in Africa – a WHO POPS/APPS project

**DOI:** 10.1186/2047-2994-4-S1-P250

**Published:** 2015-06-16

**Authors:** J Storr, C Kilpatrick, S Syed, J Dixon Hightower, D Pittet, Private Organizations for Patient Safety

**Affiliations:** 1 Service Delivery and Safety, WHO, UK; 2 Infection Control and WHO Collaborating Centre on Patient Safety, University of Geneva Hospitals, Geneva, Switzerland

## Introduction

The availability of alcohol-based handrub (ABHR) and its component parts to enable reliable use in health care are variable around the globe.

Three initiatives from the World Health Organisation (WHO) have addressed this inequity. The WHO guidelines on hand hygiene (2009) promote the use of ABHR as an easy and effective way to ensure clean safe hands at the point of care. WHO Private Organisations for Patient Safety (POPS) harnesses industry strengths to align and improve implementation of WHO recommendations, to hand hygiene in the first instance. WHO African Partnerships for Patient Safety (APPS) focuses on supporting safer healthcare delivery in hospitals with hand hygiene as a linchpin for safe quality care.

In 2013, a one-off project was launched through POPS to provide empty bottles to APPS hospitals so that operational barriers to implementation of locally produced ABHR could be addressed.

## Objectives

1. To describe an implementation-focused project spanning three WHO programs responding to locally identified barriers

2. To describe the process involved in providing a short-term solution to the identified problem

3. To systematically identify barriers and recommendations to the problem

## Methods

A tripartite approach was employed involving an open call to POPS companies, a targeted call to APPS hospitals that had previously completed a WHO training program on local production of ABHR, and brokering of the interaction between POPS and APPS via the WHO APPS team. A preparation, logistics and communications plan was adopted.

## Results

One company provided 27 990 bottles to 6 hospitals in 5 countries in Africa. It took 6 months from the start of the project until final delivery of all hardware to the hospitals. Key challenges related to mode of transportation, barriers to entry, storage and identification of reliable routes from port to hospital. Acknowledgement was received by hospital managers regarding the impact on ABHR availability at point of care.

## Conclusion

This project allowed the logistics process to be scrutinized and lessons are informing the next stage of work to address barriers. Availability of ABHR and other required resources in the African region continues to be constrained and action to address this inequity remains a key priority for WHO and POPS informed by this project.

## Disclosure of interest

None declared.

